# Nanocarriers for anticancer drugs: Challenges and perspectives

**DOI:** 10.1016/j.sjbs.2022.103298

**Published:** 2022-04-22

**Authors:** Amany I. Alqosaibi

**Affiliations:** Department of Biology, College of Science, Imam Abdulrahman Bin Faisal University, P.O. Box 1982, 31441 Dammam, Saudi Arabia

**Keywords:** Nanocarrier, Nanosystem, Nano formula cancer therapy

## Abstract

Cancer is the second most common cause of death globally, surpassed only by cardiovascular disease. One of the hallmarks of cancer is uncontrolled cell division and resistance to cell death. Multiple approaches have been developed to tackle this disease, including surgery, radiotherapy and chemotherapy. Although chemotherapy is used primarily to control cell division and induce cell death, some cancer cells are able to resist apoptosis and develop tolerance to these drugs. The side effects of chemotherapy are often overwhelming, and patients can experience more adverse effects than benefits. Furthermore, the bioavailability and stability of drugs used for chemotherapy are crucial issues that must be addressed, and there is therefore a high demand for a reliable delivery system that ensures fast and accurate targeting of treatment. In this review, we discuss the different types of nanocarriers, their properties and recent advances in formulations, with respect to relevant advantages and disadvantages of each.

## 1 Introduction

Nanomedicine is a newly emerging field that has gained significant attention worldwide. The technology in this field allows for the deeper exploration of cellular compartments, and may help to combat several diseases, including cancer (Mushtaq, 2021). The development of therapeutic nanomedicine-delivery systems represents a challenge for research groups worldwide (Bamburowicz-Klimkowska et al., 2019, Bastiancich, 2019), and there is high demand for a system that combines active functional drugs with an efficient delivery vehicle. The conventional strategy for chemotherapy delivery has several issues, including potential development of multidrug resistance, which impairs reliable use of these drugs (Karpkird, 2020, Ruman, 2020). Furthermore, clinical response to classical nanocarriers often differ from those expected from anticancer therapy. New materials and smart drug delivery platforms that respond to stimuli have therefore been developed (Elzoghby, 2020, Heinrich et al., 2020, Parayath, 2020). Several clinical trials have shown that these smart drug delivery systems enhance the therapeutic efficiency of the treatments for several types of cancers, in comparison to free drugs (Vergallo, 2021). The development of new nanocarriers capable of efficiently delivering specific drugs to their target sites is therefore required (Ruman, 2020).

**Fig. 1:**
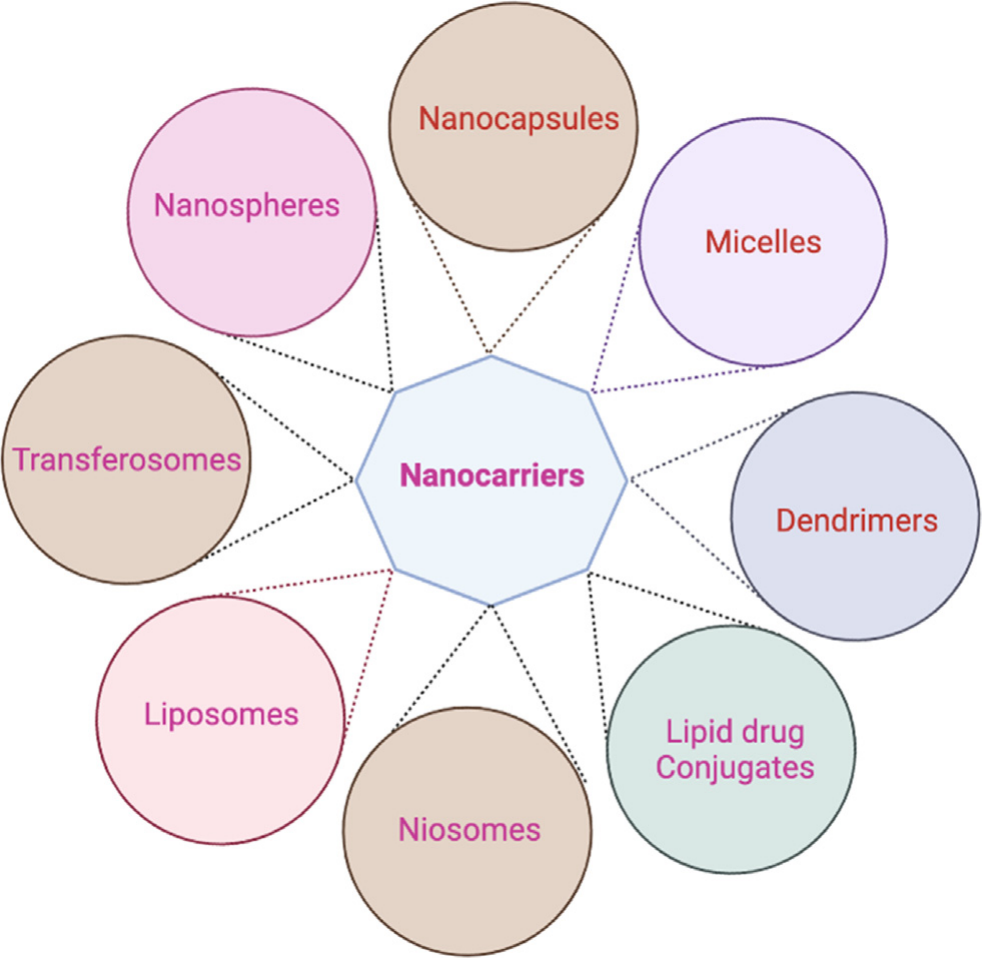
Different types of nanocarriers used to deliver drugs.

Drug delivery systems are a powerful strategy for improving the therapeutic properties of anticancer drugs (Fig. 1). They assist in overcoming issues of drug insolubility and bioavailability that can limit their usefulness for treatment (He, 2020, Laffleur and Keckeis, 2020, Güven, 2021, Mitchell, 2021). Anticancer drugs have several major limitations, which include (but are not limited to): non-specificity, wide biological distribution, short half-life and systemic toxicity (Taghizadeh, 2015). Using nanocarriers can help to minimise these drug-related obstacles and improve the therapeutic action of drugs by allowing for delivery and controlled release at specific target sites (Sharma and Bhatia, 2021, Wang, 2021).

Nanotechnology-based drug delivery systems therefore hold a great deal of potential for the development of suitable cargos/carriers to enable effective and efficient delivery of drugs against several diseases, including cancer. Furthermore, these delivery systems could provide a solution for other drug-related issues, including side effects and therapeutic efficacy (Zheng, 2020, Aranda-Lara, 2021).

Drug delivery systems are mainly pharmacological cargos. They include nanoemulsions, nanoliposomes, nanohydrogels, nanoparticles (NPs) and nanofibers, and the ability of these molecules to entrap or retain drugs and transport them to target sites has attracted the attention of the medical field (Zheng, 2020, Tomeh and Zhao, 2020, Baveloni, 2021, Hasani et al., 2021). Generally, the cargo enclosed within nanocapsules are nanoparticles (less than 100 nm in size). Their small size allows them to reach specific cellular compartments, where they deposit their cargo (Güngör and Kahraman, 2021, Martinelli, 2021). Encapsulated polymers may also be used successfully as delivery systems, and are able protect drugs from environmental factors that could cause gastrointestinal (GI) degradation (Al-Hashimi, 2021, Calvino, 2021).

Natural polysaccharides (NPLS) are the most widely available polymers, and their diverse properties give them an advantage over synthetic polymers (Niu, 2021). NPLS are also considered safe for the targeted delivery of drugs due to several unique properties, including non-toxicity, non-reactivity and biocompatibility (Shah, 2020, Chen, 2021). As a result, NPLS are commonly used as excipients in pharmaceutical formulations of drugs delivered by nanocarriers. Their physical and chemical structures can be modified conveniently to suit different applications, and some are capable of resisting the enzymatic degradation processes often possessed by target cells. (Chakka and Zhou, 2020, Guo, 2020, Meneguin, 2021, Shokri, 2021).

Drugs delivered by NPLS are able to bind to the outer layer, giving them the advantage of increased stability and solubility (Salapa, 2020, Torres-Pérez, 2020). The most common potent NPLS used to manufacture nano-coated drugs are pectin, starches, gum, chitosan, alginate and cellulose (Niu, 2021, Shah, 2020). Their unique therapeutic effects are evident in the treatment of several diseases, including cancer. This review describes the challenges of using nanocarriers for the delivery of anticancer drugs, and notes future perspectives in this promising field of research.

### 1.1 Synthesis of NPs

Considerable variation exists between NPs in terms of their shape, size, structure and synthesis process. Two main methods of NP synthesis exist, and are known as the top-down and bottom-up approaches. These methods are addressed below.

### 1.2 Top-down approach

The top-down approach is also referred to as a destructive approach, whereby the bulk of the material is degraded into small chunks before generating the desired NPs (Shafey, 2020). This approach involves several techniques, such as laser ablation, thermal decomposition and mechanical milling. During NP synthesis, size, shape and charge can easily be modified by altering the reaction conditions (Lassalle and Ferreira, 2007).

### 1.3 Bottom-up approach

In this approach, NPs are built from smaller structures, such as atoms, to finally generate the desired NPs (Yang, 2014). One method commonly employed in this approach is chemical vapor deposition spinning laser pyrolysis.

### 1.4 Drug delivery systems targeting cancer cells

Targeted drug delivery systems are a recently developed method for efficiently delivering specific drugs to targeted tumour cells (Alshememry et al., 2017, Bokan et al., 2019), and have several advantages and disadvantages. Advantages include the protection of normal cells from drug action due to the specific targeting of cancerous cells, a significant reduction in drug side effects and a decrease in drug resistance rates in some tumour cells (Ahlawat et al., 2018, Ajithkumar and Pramod, 2016). It is critical that the drugs are delivered to their target site in sufficient quantities to exert their functions, and this should be considered when choosing and designing drug delivery systems. The nucleus of the cell is the primary target site for most drugs. Although the design of NPs allows them to enter the cytosol, there is no guarantee that they will be able to reach the nucleus. There are therefore two approaches for targeting, described below.

### 1.5 Active targeting

In active drug targeting, drugs can be delivered using pretentious structures, such as antibodies or peptides, to access receptors and target sites. Active targeting includes techniques such as ultrasonic energy and magnetic fields. The active targeting system is comprised of three components: a ligand that serves as a targeting moiety, a polymer to act as a carrier and the specific drugs of interest. The antigen functions as an active targeting system, as it is expressed only on tumour cells. This allows internalisation of the system via receptor-mediated endocytosis (Kumar Khanna, 2012). Active drug targeting methods are crucial for delivering not only drugs, but also genes, proteins and theranostics to their target sites, sparing the normal cells from exposure and thus decreasing potential side effects. This system also increases the quantity of drug delivered compared to ordinary drug delivery systems.

When accumulated in the interstitial space surrounding the tumour cells, the drug can become entrapped in the active mechanisms of the cells. This occurs due to the decoration of the nanosystem with pretentious motifs that attach to proteins overly expressed on the tumour cell’s surface. This decoration improves the chemical and physical affinity of the nanosystem for the surface of the cancer cell (Attia, 2019). Target sites are molecules to which the nanosystem attaches before entering the cell. These include transferrin receptors that are used to reach the brain tumour microenvironment, and transferrin ligands have been used to target glioma cells by loading them onto solid lipid NPs (Jain, 2013), dendrimers (He, 2011), micelles (Zhang, 2012) and superparamagnetic iron oxide NPs (Jiang, 2012).

### 1.6 Passive targeting

Direct drug injection into the bloodstream is a typical example of passive drug targeting (otherwise known as physical targeting). In this mechanism, drugs are prepared to minimise their removal by body mechanisms such as metabolism, excretion, opsonization and phagocytosis. It is well-documented that blood vessels become more permeable under certain conditions, including hypoxia and inflammation (Torchilin, 2011). Tumour cells often establish new blood vessels when they are actively dividing, through the process of angiogenesis. This renders the newly formed blood vessels more permeable, and allows macromolecules larger than 40 kDa (including nanosystems) to pass through. The encapsulation of small-sized drugs into nano form can therefore enhance their pharmacokinetics and reduce potential side effects. This type of targeting is referred to as passive targeting, as it relies on the use of nanocarriers (Maeda, 2001, Fang et al., 2011).

It should be noted, however, that hypoxic and necrotic lesions occurring in the core part of the tumour mass can prevent nanocarriers and nanomedicine from accessing the entire tumour. These necrotic lesions result from insufficient delivery of oxygen and nutrients to these regions, due to their distance from the main tumour blood supply. Furthermore, the interstitial pressure acting on the blood vessels in the core part of the tumour mass makes them less permeable than those in the periphery (Fang et al., 2011, Kobayashi et al., 2013).

### 1.7 Drug nano-delivery systems

Interestingly, NPLS are functional macromolecular biopolymers generally isolated from several origins, including plants (pectin and cellulose), animals (chitosan), bacteria (xanthan, gum and dextran) and algae (alginate). These polysaccharides are commonly composed of more than ten monosaccharide units that are linked by glycoside in linear or branched chains, and have a high profile of safety, stability and biocompatibility. They can easily be modified and manipulated according to the required designs and structures for further application in many fields, including biomedical and pharmaceutical research. NPLS are used effectively in biomedical research due to their various biological properties, including their hypoglycaemic, anticancer, and anticoagulant effects.

**Fig. 2:**
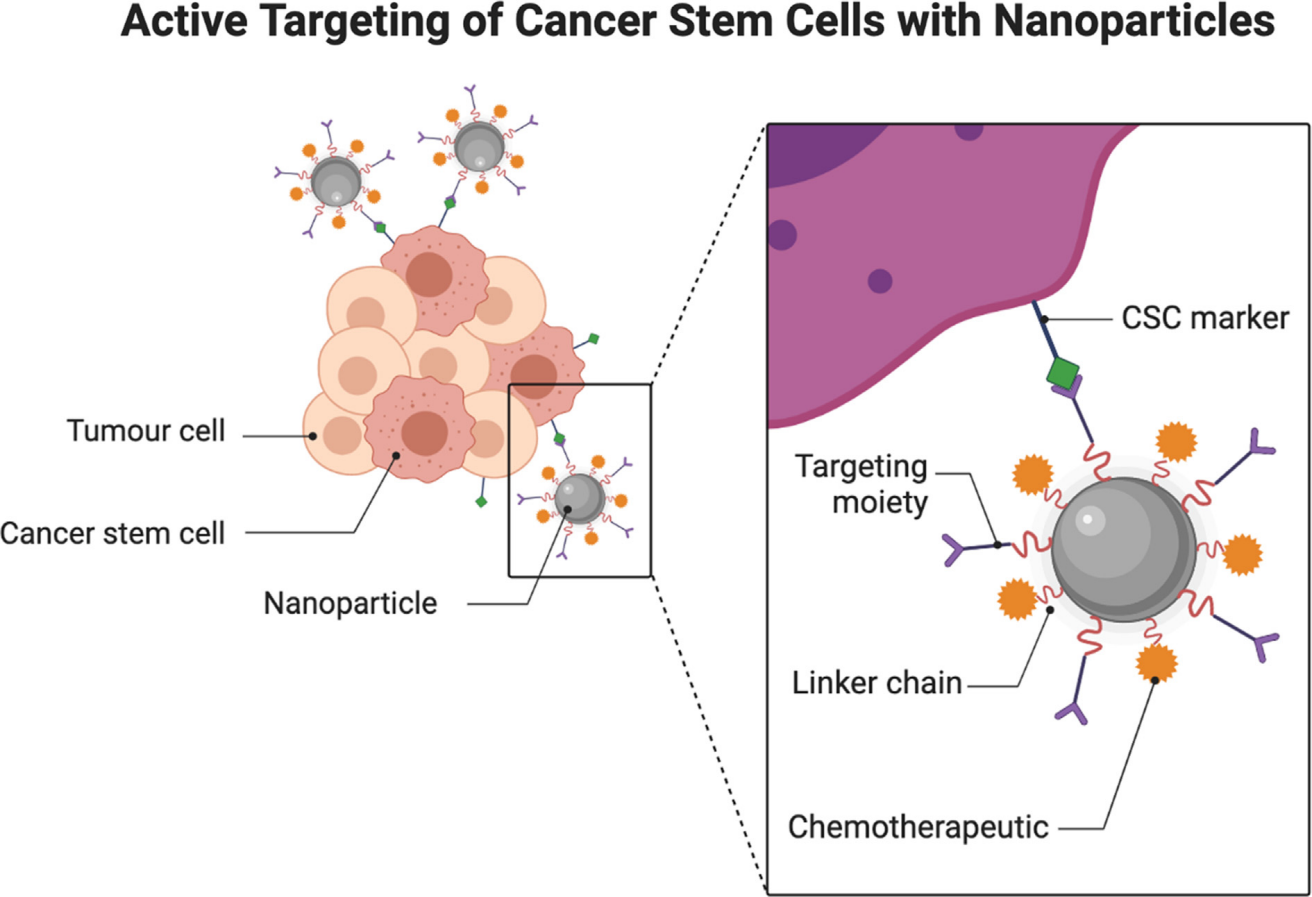
Interaction between nanoparticles carrying drugs and the cell membrane.

NPLS are used as candidate excipients for drug nano-delivery systems due to the presence of specific ligands, which bind to target cell receptors. These ligands are involved in the controlled and targeted transport of the drugs for delivery. In addition, NPLS are generally biodegradable, biocompatible and have lower toxicity levels (Fig. 2).

**Fig. 3:**
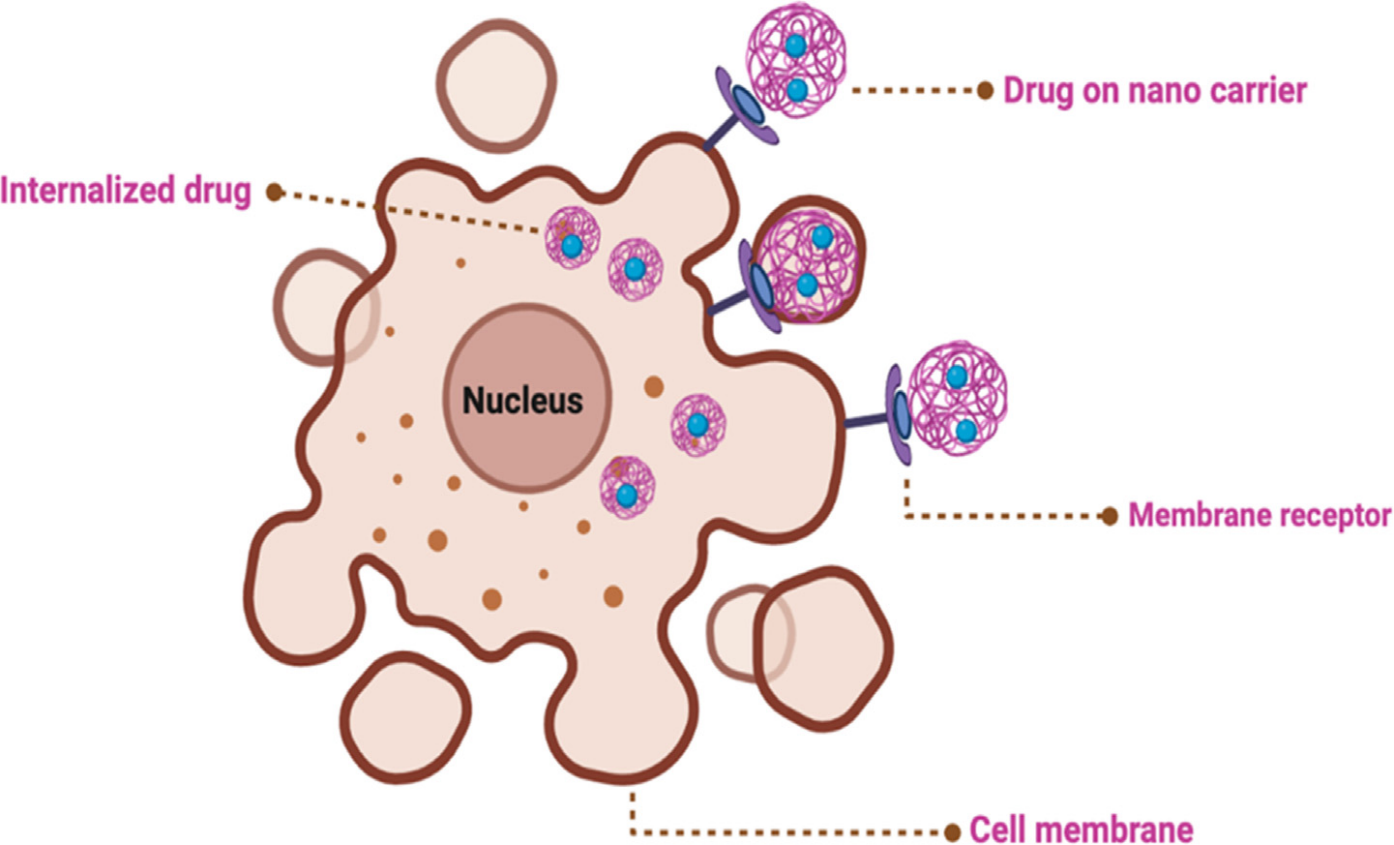
The active sites on the cell membrane and the internalisation of the target drug into cancer cells.

NPLS can be readily chemically or biochemically modified to produce a variety of active forms with multiple functions, due to their surface structure that contains functional groups such as carboxyl, hydroxyl and amino groups, In particular, these derived groups play an instrumental role in the formation of non-covalent bonds associated with living muscles, including mucous membranes and the epithelium, that are involved in bio-docking. For example, the NPLS alginate, pectin, starch and chitosan are potent bio-adhesive agents (Fig. 3).

When bio-adhesive agents are used as drug delivery systems, cellular/drug uptake is enhanced successfully. These prominent advantages of NPLS make them a method of choice for the delivery of chemotherapeutic drugs, particularly for cancer treatment.

Recently, NPLS have been studied extensively to gain insight into their use as stabilisers in various nanocarriers formulations (Rehman, 2020, Gheorghita Puscaselu, 2020, Pejin et al., 2014). Biopolymers (such as polysaccharides) are used as effective nanocarriers for drug delivery due to their high biocompatibility and low immunogenicity (Alshememry et al., 2017).

The different types of NPLS are discussed in more detail in the following sections.

### 1.8 Chitosan

Chitosan is a well-known linear cationic hetero-polysaccharide consisting of β-1,4-linked 2-amino-2-deoxy-glucopyranose and 2-acetamido-2-deoxy residues-β-D-glucopyranose. It is one of the most prominent natural polysaccharides and is widely used in nano-drug delivery systems, due to its biocompatibility and biodegradability (Jhaveri, 2021, Narmani and Jafari, 2021). Chitosan has several properties that make it an efficient nanocarrier, including high stability levels in serum, low immunogenicity, lengthy circulation time, biocompatibility and high bioavailability (Mushtaq, 2021, Narmani and Jafari, 2021, Pejin, 2014).

Chitosan is extracted from crustaceans and used for various pharmaceutical and clinical applications, including as a nano-drug delivery system. The amino-functional groups in the chitosan structure make it soluble in acidic media and are crucial for the biochemical adaptations and electrostatic interactions in drug nano delivery systems.

Furthermore, the positively-charged amino groups may provide chitosan with immunogenic properties, thus enabling its use as a bio-adhesive material capable of attaching to the mucosal cell surface. The positive charge of chitosan also enables the opening of epithelial tight junctions and widening of passages through adjacent cell pathways, supporting the transport of drugs containing hydrophilic compounds into target tissues.

Chitosan has been widely used as a carrier agent for nanocarrier fabrication in drug nanosystems against various diseases, including cancer. A study conducted by Akolade et al. (2017) found that curcumin incorporated into chitosan showed an improvement in encapsulation efficiency, with a loading capacity that reached 64–76% (Akolade et al., 2017). Curcumin was also encapsulated into chitosan nanoliposomes, and showed significant loading compared to conventional structures in vaginal applications (Saesoo, 2016). Other substances have been incorporated into chitosan-coated calcium-alginate nanoparticles, including liraglutide, which exhibited an encapsulation efficiency of more than 925 and was found to be very effective in diabetes (Shamekhi et al., 2018). Chitosan-based nanoparticles have been used against several bacterial (Rashki, 2021, Abd El-Hack, 2020) and viral infections, such as coronavirus (CoVs) (Rasul, 2020). When loaded with glycol, they have been found to accumulate inside tumour cells, suggesting that they are modifiable for use in other applications besides the drug delivery (Ryu, 2020). Furthermore, chitosan was combined with a programmed death-ligand 1 (PD-L1) to enable efficient transmucosal delivery of this protein involved in programmed cell death (Jin, 2021), and was also used in combination with carbon quantum dot and DNA aptamer for specific delivery of 5-fluorouracil (5-FU) in breast cancer cells. The success of this approach was determined by measuring the high expression profile of different apoptosis-related genes, such as *BAX and BCL-2* (Zavareh, 2020).

Chitosan-raloxifene was first introduced by Mohammadi et al. as a novel targeting therapy for breast cancer, and was able to carry doxorubicin. This formulation was found to have nearly 95% drug loading capacity (60% if the drug was released with a high stability) (Mohammadi, 2020). Chitosan was also conjugated with pluronic P123 polymers to target lung cancer cells, and this conjugated formula significantly enhanced cancer cell uptake, due to the high affinity between the modified liposome and the tumour cells. The same profile was observed in lung cancer mouse models, where paclitaxel loaded onto chitosan-liposome resulted in 84% inhibition of tumour growth (Miao, 2021). Many advantages are associated with the use of chitosan as a nanocarrier vehicle, including enhanced drug absorption, membrane interaction, resistance to migration, stimulation of the immune response and prolonged drug release (Nandgude and Pagar, 2021).

### 1.9 Cellulose

Cellulose is one of the most widely occurring natural polysaccharide elements. This biological compound is obtained primarily from bacteria, fungi, plants and algae (Hemmati, 2018). The structure of cellulose consists of D-glucopyranoses rings arranged in a specific manner (Bajpai, 2019).

Cellulose is regarded as a biologically compatible biomolecule with low-toxicity characterized by unique chemical and physical properties, making it useful for a wide variety of applications in medical science, including the transport of drugs to target sites. However, the original form of this biomolecule is not soluble in water, which limits its applications. Various types of modified cellulose have been created to tackle this problem and increase its applications. Several of these, such as nanofibrous cellulose, cellulose ethers, methylcellulose and hydroxypropyl cellulose, are available as nano-drug packaging and delivery for the treatment of various diseases. Due to their functional features, the reorganised forms of cellulose are used as thickeners, stabilizing agents and to manufacture nanocarriers for delivery of specific drugs.

Cellulose adsorbed with polyethylene has been used to fabricate plant-based protein-loaded nanoliposomes, which provided a controlled release of apolipoprotein in the GI tract (Patel et al., 2020). In rheumatoid arthritis, cellulose nanoparticles have been combined with sodium to deliver chloroquine for treatment as an external ointment in mouse models of the disease. The results using this delivery system showed significantly better results than the drug on its own (Bhalekar, 2015).

Cellulose is one of the most common polysaccharides in nature, and its inertness and biocompatibility make it a strong candidate as a novel nanocarrier. Several formulations have been developed based on cellulose-inducing composites, coatings and films, and have been used in nanoscale forms. These cellulose-based formulations are used in various fields, including medicine, biology, and even electronics and energy (Carvalho, 2021). Cellulose can be formulated into an elongated biobased nanostructure known as cellulose nanocrystals. It possesses novel characteristics that can treat various diseases, including cancer (Pinto, 2021).

### 1.10 Starches

Environmentally friendly nanocomposites were developed for their application as safe and biodegradable nanocarriers. These nanocarriers include starch (Li, 2019), a promising natural polysaccharide made from a biopolymer of long chains of glucose with a large molecular weight (Duan, 2020).

Recently, starch has been subjected to various modifications for different applications, including the attachment of L-alanine, L-lysine and L-phenylalanine. The spherical shape of the molecules formed by this modification is suitable for use as a nanocarrier for several drugs, including naproxen (Namazi and Abdollahzadeh, 2018).

In general, native starches have been used to enable the controlled release of drugs due to several remarkable advantages, including biocompatibility, low toxicity levels, inexpensiveness and increased drug solubility. One limitation of starch is its low emulsifying ability, which can be enhanced using modifications to improve absorption in the gastrointestinal tract. These modifications include enzymatic, chemical and physical activation (Bravo-Núñez et al., 2019). Chemically, native starch is acetylated, oxidized or hydroxypropylated to obtain a modified molecule capable of significant drug delivery (Bai and Shi, 2016, Zhu, 2017).

In recent years, the development of nanotechnology has broadened the applications of several biopolymers, including starch. For optimal use of these modified polymers, a deeper understanding of their structure at the nanoscale becomes necessary (Torres, 2019).

Starch conjugated with curcumin has recently been used at the nanoscale to deliver the latter bioagent efficiently. This composition was found to have a high drug loading capacity, enhanced colloidal stability and an acid-sensitive release profile. Furthermore, this nano formula significantly increased the solubility of curcumin compared to a free curcumin and protected it from degradation when exposed to UV rays and elevated temperatures. This formula has been used to treat several diseases, including cancer (Chen, 2020).

Cholesterol and imidazole are biomolecules that are used to oxidize starch. The end product is an oxidized starch linked with cholesterol and imidazole that is converted into nanoparticles by dialysis. This formula is used to deliver curcumin into cancer cells effectively, where loaded curcumin is released at pH 7.4 (compared to at pH 5.5 when curcumin is used alone) (Zhu, 2017).

Starch has also been used to encapsulate copper oxide nanoparticles and facilitate their target release in breast cancer cells (MDA-MB-231). Data showed that this nano formula induced cytotoxicity in cells, with an IC50 of nearly 21 μg/mL. The cytotoxicity was caused by the generation of reactive oxygen species (ROS) and the reduction of mitochondrial membrane potential (Mariadoss, 2020).

Similarly, mannose was conjugated with 5-FU and loaded onto starch nanoparticles to target liver cancer cells. When loaded, 5-FU was found to have a drug uptake efficiency of between 64.2 and 82.3%. In vivo studies indicated that mannose conjugated with NPs prolonged the plasma levels of 5-FU. Furthermore, plasma profiling showed an elevated level of 5-FU in the liver, suggesting the efficiency of this nanocarrier for delivering the drug to the target site (Jain, 2021).

Epigenetic drugs (such as histone deacetylase inhibitors CG-1521) have also been encapsulated in starch nanoparticles, to enhance their bioavailability and solubility. Data showed that encapsulation of CG-1521 reduced release rates and increased drug cytotoxicity in breast cancer cells MFC-7. This nano formula induced cell cycle arrest and apoptosis, suggesting that encapsulating this drug in a starch nanocarrier can significantly improve its delivery (Alp, 2019).

### 1.11 Pectin

Pectin is a naturally occurring biopolymer with linear polysaccharides composed of α-(1–4)-D-galacturonic acid, and shows promise for use in different applications, including drug delivery (Rehman, 2019). Pectin is not digestible in the gastrointestinal tract, where its solubility is reduced. It is, however, absorbed in the colon through the action of pectinolytic enzymes produced by commensal bacteria (Shishir, 2019).

Pectin is characterized by its hydrophobicity, a property that makes it useful as a nanocarrier, and it has thus been used in the transdermal administration of some drugs. Pectin has also been used to synthesize drug-loaded nanocarriers in the form of nanoparticles, nanoemulsions and nanoliposomes. Several drugs have been loaded onto pectin nanocarriers, including curcumin (Alippilakkotte and Sreejith, 2018), cefazolin (Shahzad, 2019) and metformin (Chinnaiyan et al., 2019).

Cancer cell resistance limits the efficacy of chemotherapeutic drugs. This problem can be tackled by using essential oils, though the low bioavailability remains a crucial factor. Thus, nano emulsifying agents represent a promising tool for overcoming this obstacle. One study combined citrus-pectin with Zataria multiflora essential oil to determine its antiproliferative properties. This preparation was found to induce apoptosis in breast cancer cells by triggering activation of ROS, loss of mitochondrial membrane potential and arrest of the cell cycle at the G2/M phase (Salehi, 2020).

Due to its self-healing properties, composite hydrogels were synthesized from oxidised pectin, chitosan and gamma-Fe2O3 to deliver drugs into target cells. This formula was found to have high solubility and biocompatibility, as well as self-healing and anti-proliferative properties, particularly when loaded with 5-FU, which the composite was able to release continuously over 12 h. (Li, 2020).

Pectin can also be conjugated with chitosan for improved delivery efficiency. One study developed pectin-chitosan nanoparticles loaded with cetuximab, and found that the cellular uptake of this formula was enhanced in colon cancer cells. This resulted in reduced cancer cell propagation and the arrest of the cell cycle at the G2/M phase, suggesting that this nano formula has antiproliferative properties (Sabra et al., 2019).

In addition to its modified forms, pectin shows promise for tumour-targeted drug delivery. Several studies have investigated the combination of pectin nanoparticles with nanoparticles of different metals (such as gold) for use as anti-cancer treatment for breast cancer cells (Suganya, 2016). Increased apoptosis and the results of comet assays suggest that this combination is effective as an antiproliferative nano formula (Suganya, 2016).

### 1.12 Alginates

Alginates are linear anionic water-soluble polysaccharides that commonly occur naturally in brown seaweeds. Alginates have divalent cations, which is advantageous in the synthesis of nano-drug delivery systems. Their safety and biocompatibility have made them useful for the encapsulation of several drugs. The presence of cations (such as calcium ions) makes alginates good nanocarriers, and allows them to be used in the fabrication of hydrogels.

The process of transporting drugs using hydrogels relies mainly on pH, and drugs encapsulated by alginates will not be released in the acidic environment of the stomach. As the compound reaches the intestine and the pH increases, drug release is allowed to commence. Alginates can be used as delivery tools for anticancer drugs due to their low toxicity and high biocompatibility and biodegradability (Jayapal and Dhanaraj, 2017). They can also be combined with other natural polysaccharides, such as chitosan and pectin.

Many studies have indicated the potential use of alginate NPs-based platforms for the successful delivery of some anti-tumour drugs (He, 2020). In one study, curcumin was loaded onto magnetic alginate/chitosan NPs to improve its bioavailability and cytotoxicity in breast cancer cells. The results indicated that cells treated with loaded curcumin had 3–6 times more efficient drug uptake, suggesting that this nanocarrier provides a reliable method of drug delivery (Song, 2018).

In another study, doxorubicin (a common chemotherapeutic drug) was loaded onto alginate nanohydrogels, and this formula was found to produce sustained release of doxorubicin in tumour cells (Zhou et al., 2018). Other researchers loaded insulin onto alginate nanoparticles to treat diabetic rats, and found it reduced glucose levels by 40% for more than 18 h (Sarmento, 2007).

For evaluation as a breast cancer treatment, the oral chemotherapy drug exemestane was loaded onto alginate NPs. Results of in vitro experiments indicated that alginate nanoparticles (ALG-NPs) serve as a reliable drug delivery system in breast cancer (Jayapal and Dhanaraj, 2017). In another study, cisplatin was co-loaded onto alginate hydrogel and gold NPs for delivery; the combination was found to produce higher levels of tumour inhibition (Mirrahimi, 2020).

Researchers have also shown that catechol-functionalised alginate NPs can be used for the treatment of bladder cancer. This nano formula was fabricated using coupling chemistry, and in vivo analysis showed improved retention in the mucosa of porcine bladder tissue compared to the unmodified form. Additionally, this combination also showed high loading efficiency and capacity. This nano formula can therefore serve as a mucoadhesive nano agent for the treatment of bladder cancer (Sahatsapan, 2020).

Curcumin glutaric acid is the precursor of the active form curcumin, and is characterized by high solubility compared to the active form. To enhance these properties, curcumin glutaric acid was encapsulated into chitosan/alginate NPs. This nano formula showed a high rate of cellular uptake in colorectal cancer cells, and had improved antiproliferative activity compared to the free form. Oral administration of this nano form could therefore be used for the treatment of some cancer types (Sorasitthiyanukarn, 2018).

One study loaded doxorubicin into alginate/chitosan in nano form, according to the water-in-oil emulsification principle. The efficacy of this novel formula was evaluated in murine breast cancer cells, and the results showed that the nano form had an IC50 of 0.15 μg/mL, compared to free doxorubicin (0.13 μg/mL), suggesting that the nano formula was more efficient in delivering the cytotoxic drug (Rosch, 2019).

### 1.13 Hyaluronic acid

Hyaluronic acid (HA) is a naturally occurring polysaccharide found in all connective tissues of animals and humans. Researchers conjugated camptothecin with an HA shell, and found that this formula was capable of eliminating primary breast cancer cells in mice (Sun, 2019). Another study investigating the treatment of CD44- expressing breast cancer cells prepared a combination of HA, chitosan and lipoic acid NPs. The effectiveness of these combinatory NPs was determined using MTT, TUNEL, CASP3 and Rhodamine 123 assays. The combination showed sustained release, high cellular internalization and rapid drug release inside breast cancer cells (MCF-7). These data suggest that this nano formula could be a promising platform for targeting the delivery of cancer-treating drugs (Rezaei, 2020).

HA-based polymeric NPs have also been used to encapsulate IR780 for breast cancer treatment. This nano formula was co-encapsulated with doxorubicin to enhance its therapeutic activity. Two-dimensional and in vitro analyses indicated a higher cellular internalization of the nano formula by breast cancer cells compared to non-malignant cells. These results demonstrate the effectiveness of this nano formula for delivery of cancer-treating drugs (Alves, 2019).

Docetaxel, a commonly used chemotherapeutic drug, is inactivated by the action of CYP1B1, an enzyme overexpressed by MDR breast cancer cells. HA, combined with polyethyleneimine in nano form, was loaded with docetaxel and α-napthtoflavone (an inhibitor of CYP1B1) to overcome the MDR caused by CYP1B1 in breast cancer cells. This formula could reverberate MDR by downregulating CYP1B1 in cancer cells, thus enhancing the therapeutic activity of docetaxel (Zhang, 2019).

The delivery of chemotherapeutic drugs encounters several obstacles, including a lack of specificity to target cells, low rates of cellular internalisation and higher rates of hepatic accumulation. To address this problem, researchers prepared a composite nano formula of HA-paclitaxel-methoxy polyethylene glycol, and tested it in lung cancer cells expressing CD44. This formulation was found to have reduced hepatic accumulation compared to the free drug form, and enhanced antitumour activity in vivo. This composite nano formula can therefore be applied directly for the improvement of delivery of cancer-treating agents both in vitro and in vivo (Luo, 2020).

5-FU is a commonly used anticancer drug with limited bioavailability. In their study, Mansoori et al. developed HA-modified liposomes to deliver 5-FU into CD44-expressing breast and colorectal cancer cells. Apoptosis assays indicated that treated cells were arrested at the G0/G1 phase of the cell cycle. Furthermore, their results showed that colony formation was reduced when given 5-FU-HA and 5-FU-lipo, compared to the free drug (Mansoori, 2020).

HA has also been used to modify Au-Ag alloy NPs to target CD44-expressing 4 T1 breast cancer cells specifically. The peroxidase-like activity of the alloy helps to generate more OH and release Ag^+^ at the tumour site, resulting in the efficient therapeutic action of the alloy (Chong, 2020).

Quinacrine is primarily used in the treatment of malaria, but has also been found to have anticancer properties. The main challenge of quinacrine is its hepatotoxicity; to overcome this, a nanocomposite was prepared using HA and lactoferrin to target pancreatic cancer cells (PANC-1). The composite yielded a 3-fold decrease in the IC50, indicating its potent anticancer activity. In vivo studies suggested that the composite reduced tumour volume and improved the survival of rats, without any adverse effects on vital organs (Etman, 2020).

Recently, raloxifene has been used in the treatment of lung cancer and prevention of hepatic cancer. Raloxifene resistance remains a significant obstacle to the use of this drug. To address this issue, researchers loaded raloxifene into HA and chitosan nanoparticles to increase the drug’s half-life and enhance its release. This formulation was tested on non-small cell lung cancer (A549) and hepatocellular carcinoma (HCC) cells, and the results showed that it had higher drug uptake capacity than the free drug alone. This was the first study to assess the loading efficiency of raloxifene in HA and chitosan NPs (Almutairi et al., 2019).

HA has been shown to be useful when combined with gold nanoparticles (AuNPs) in the preparation of docetaxel for the treatment of cancer cells. This combination demonstrated greater cytotoxicity and inhibition of cancer growth in the tumour model, compared to free docetaxel (Gotov et al., 2018).

The drug loading properties of zirconium phosphate and cancer cell targeting abilities of HA were combined by researchers by adding HA to zirconium phosphate NPs. This system was then loaded with paclitaxel as an antiproliferative drug to target lung cancer cells. The loaded drug showed increased accumulation inside target cells compared to the free drug alone, and in vivo analysis demonstrated increased anticancer activity (Li et al., 2017).

### 1.14 Dextran

Researchers combined dextran with chitosan NPs, and loaded this with NIK/STAT3-specific siRNA and BV6 to induce cell death in different cancers, including breast and colorectal cancer. The results indicated that this combination induced apoptosis, inhibited cell proliferation and reduced cellular migration, angiogenesis and colony formation of tumour cells (Nikkhoo, 2020).

The anti-HER2 antibody is a promising targeting molecule for tumour-specific cancer treatment. In one study, this antibody was conjugated with dextran and spermine in a nano form, and the efficiency of this formula against cancer cells was evaluated. The results indicated that the conjugated antibody had no toxic effect on SKBR3 and human fibroblast cells. When used in hyperthermia treatment, the conjugated anti-HER2-spermine-dextran NPs were found to be able to target and induce apoptosis in the studied cells (Avazzadeh, 2017).

To increase the efficiency of 5-FU and paclitaxel, researchers loaded these molecules onto chitosan/dextran NPs to facilitate dual drug delivery. In vitro analysis revealed that the formula exhibited a controlled drug release. The cytotoxic effect of this nano formula on hepatocellular carcinoma cells (HepG2) was evaluated, and the results showed that the dual drug inhibited cell proliferation and induced apoptosis. Moreover, cellular uptake analysis indicated high drug internalisation of both paclitaxel and 5-FU in liver cancer cells (Wang, 2020).

In another study, researchers loaded methotrexate onto a dextran-curcumin nano-system to facilitate effective delivery into breast cancer cells (MCF-7). Their findings suggested that the nano-system was able to control methotrexate release and produced high rates of drug internalisation in these cells (Curcio, 2019).

Further studies have been performed investigating the use of dextran for improving drug delivery. A combination of dextran and folic acid-coated superparamagnetic iron oxide was used to enhance the targeting efficiency and drug uptake of vinblastine in pancreatic cancer cells. The synthesized nanocarrier was spherical in shape, and the system was found to activate PDL1, Casp3 and NF-1 in pancreatic cancer cells, which, in turn, induced apoptosis and reduced cell proliferation (Albukhaty, 2020).

To overcome the resistance to doxorubicin commonly acquired by cancer cells (particularly in breast cancer), doxorubicin was encapsulated into dextran-chitosan NPs. This formula showed higher cytotoxicity in cancer cells, and produced no effect on normal cells. Furthermore, this nano formula enhanced drug uptake and induced apoptosis by increasing the Bax/Bcl-xL ratio, and upregulating p53 and p21 (Siddharth, 2017).

Dextran was also used in combination with polyethylamine and iron oxide NPs as part of a magnetic gene carrier to deliver miR-320b into osteosarcoma cells. The generation of a magnetic field enabled this nano formula to enter OS cells in nude mice, and revealed the anti-osteosarcoma effects of miR-320b, which likely reduces proliferation by regulating the Hippo pathway through YOD1 (Gong, 2020). Dextran-coated magnetic NPs were also used to efficiently and selectively deliver miR-29a into breast cancer cells, where it led to the downregulation of several anti-apoptotic genes (Yalcin, 2019).

Capecitabine is an antineoplastic drug that can be loaded onto a cationic dextran-spermine polymer for effective delivery into glioblastoma cells. Cytotoxicity data showed that the loaded drug had increased toxicity and enhanced glioblastoma uptake compared to the free drug (Ghadiri, 2017).

Controlled drug release of 5-FU has been demonstrated by loading it onto colonic enzyme-responsive dextran-based oligoester NPs. The enzyme dextranase triggered the release of the encapsulated drug, and the actions of this enzyme were likely the cause of the increased bioavailability and elevated toxicity of the nano formula for the treatment of colorectal cancer (Abid, 2020).

Dextran has also been coated with Goldman NPs for delivery of doxorubicin into liver cancer cells. The nano formula exhibited a high capacity for prolonged doxorubicin release, which enhanced the therapeutic action of the drug. It also decreased the adverse effects of doxorubicin, and inhibited the growth and proliferation of liver cancer cells. This formula therefore represents a promising step for the treatment of cancer by improving drug delivery (Li, 2018).

Dextran sulphate-chitosan NPs have been used to deliver lapatinib into cancer cells, and cytotoxicity assays indicated that loaded lapatinib demonstrated potent anticancer activity following 48 h of treatment, in comparison to the drug alone. These results suggest that the nanocarrier efficiently releases the hydrophobic drug in a controlled manner (Mobasseri, 2017).

The most significant issues with paclitaxel are its low solubility and bioavailability. Bakrania et al. (2018) demonstrated that these problems could be resolved when paclitaxel was delivered in a DEAE-dextran nano formula into triple-negative breast cancer cells. Their results showed that encapsulated paclitaxel was more potent for inhibiting ROS via β-interferon induction. Moreover, the nano formula was also found to inhibit VEGF and NOTCH1 overexpression. This formula therefore demonstrated total anticancer activity against TNBC (Bakrania, 2018).

## 2 Conclusion

The use of nanocarriers for drug delivery provides the possibility of treatment for several diseases, including cancer, that have long been considered untreatable. These nano formulations enhance the bioavailability, solubility and uptake of drugs, thus reducing treatment side effects while increasing cytotoxicity. This review sheds light on several types of nanocarriers, with particular focus on biopolymers and their potential for drug delivery in cancer therapy. Future research should focus on the development of new generations of nanocarriers to facilitate the delivery of the increasing number of drugs targeting different diseases, including cancer.

## Declaration of Competing Interest

The authors declare that they have no known competing financial interests or personal relationships that could have appeared to influence the work reported in this paper.
